# Lives of the Rohingya children in limbo: Childhood, education, and children's rights in refugee camps in Bangladesh

**DOI:** 10.1007/s11125-022-09631-8

**Published:** 2023-05-03

**Authors:** M. Mahruf C. Shohel

**Affiliations:** University of Roehampton, Roehampton Lane, London, SW15 5PJ UK

**Keywords:** Childhood, Children’s rights, Education, Displaced Rohingya children, Rohingya crisis, Refugee camps, Bangladesh, Myanmar

## Abstract

The Rohingyas, an ethnic minority of Myanmar, have been denied human rights, including nationality. For decades, they have suffered from brutal oppression, discrimination, violence, torture, unjust prosecution, murder, and extreme poverty. Hostile situations in Rakhine State have forced the Rohingyas to flee from their homes and seek refuge in neighboring Bangladesh and other countries, including India, Thailand, Sri Lanka, Nepal, Pakistan, Malaysia, Indonesia, and even distant Saudi Arabia. Many of the Rohingya refugees are children who escaped from their homeland with traumatic experiences and memories. In Bangladesh, the Rohingya children live in desperate conditions in overcrowded, makeshift refugee camps. They are exhausted, frustrated, and poorly nourished, and they have been battling diseases, including Covid-19, as their conditions have become more challenging and volatile. This article explores the historical context of this crisis and analyzes, from the human rights perspective, issues associated with the Rohingyas’ displacement and the impact it has had on the Rohingya children.

The Rohingyas, an ethnic minority of Myanmar, are one of the most marginalized and systematically persecuted communities in recent history (Chaudhury et al., 2018; Zarni & Cowley, 2014). Research has found evidence of sexual violence, brutal torture, and ethnic cleansing directed toward the Rohingyas in Rakhine State (also known as Arakan) (Hutchinson, 2018; Shohel, 2022). Since 1982, the government of Myanmar has denied Rohingyas’ citizenship (Ware & Laoutides, 2018). As stateless people, they have been compelled to leave their homes and are termed “forcibly displaced refugees” (FDRs) or “forcibly displaced Myanmar nationals” (FDMNs). Since 1948, most Rohingyas have sought refuge in Bangladesh (Bhatia et al., 2018) (see Figure 1). During the last influx, in 2017, over 655,500 Rohingya refugees entered Bangladesh (ISCG, 2018), 58% of whom were children under 18 (UNICEF, 2017). According to recent data, 950,972 registered Rohingya refugees (Rohingya refugees registered under the joint Government-UNHCR registration exercise as of 30 November 2022) live in refugee camps in Bangladesh (DGHS, 2022). Although the Rohingya refugees are predominantly Muslim, there are Hindu Rohingyas, too, who have fled to Bangladesh in recent years (Mithun & Arefin, 2020).

The Rohingya refugee crisis is a “complex emergency”. According to WHO, complex emergencies are situations of

**Fig. 1:**
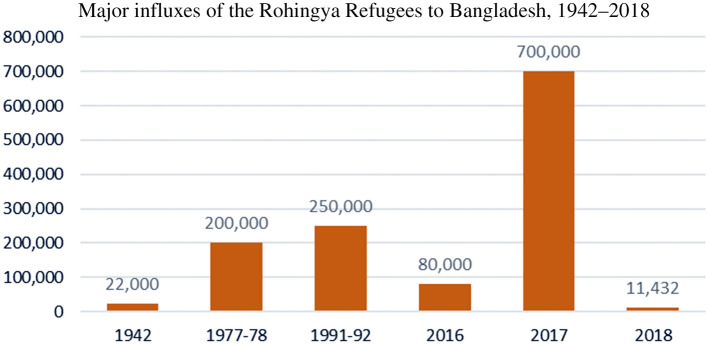
Major influxes of Rohingya refugees to Bangladesh, 1942–2018

disrupted livelihoods and threats to life produced by warfare, civil disturbance, or large-scale movements of people, in which any response has to be conducted in a difficult political and security environment. However, the Rohingyas have struggled constantly to meet their most basic needs since leaving Myanmar, yet it is almost impossible for the responsible authorities to ensure their human rights in the camps in Bangladesh (International Rescue Committee, 2019). For the Rohingya children in these camps, food, shelter, health, well-being, safety, and education are key concerns (Shohel, 2022; Shohel et al., 2023a & b). Coping with fear and uncertainty is not easy for the Rohingya children. Since arriving in the camps, they have been exposed to many dangers, including measles, cholera outbreaks, the Covid-19 pandemic, poor sanitation, food insecurity, weather hazards, and fire (Ahmad et al., 2020; Guglielmi et al., 2020; Hassan et al., 2018; Landry & Tupetz, 2018; Pocock et al., 2017). These factors have contributed to new cases of malnutrition as well as to the deteriorating health of those already battling undernourishment.

Many Rohingya families do not eat well enough or frequently enough to remain healthy and fit. The poor quality and diversity of their food threaten the development, growth, and even survival of the Rohingya children (Jean, 2020). During the Covid-19 pandemic, around 315,000 Rohingya refugee children and teenagers were freed from their educational institutions in the refugee camps in Bangladesh (UNICEF, 2020). The mother of a 5-year-old Rohingya child expressed her anxiety about the situation:

“We are afraid because we fled from Myanmar to save our lives. If our children die, what will happen to our people?” (Jean, 2020).

Interviews with Rohingya children in Bangladesh have shown they do not feel safe in the camps and, due to the unhealthy conditions, are vulnerable to disease (Plan International, Save the Children International, & World Vision International, 2018). Over 11% of children in the camps suffer from acute malnutrition, and more than 30% suffer from chronic malnutrition (Jean, 2020). Studies show that the prevalence in the camps of GAM and anaemia exceeded the global emergency thresholds of 15% and 40%, respectively, regardless of length of stay (Leidman et al., 2018) and that, due to the lack of food, health services, medicine, and sanitation, those who reside in the camps experience poor health, malnutrition, waterborne illness, and a lack of obstetric care (Mahmood et al., 2017). The Rohingya refugee children, especially girls, are very vulnerable to violence and trafficking both for sex and manual work (Babu, 2020; Shohel, 2022).

Rakhine State (see Figure 2) is situated on the west coast of Myanmar and is one of that country’s least developed regions, characterized by extreme poverty, low incomes, and poor infrastructure and public services. The situation is worsened by natural calamities such as cyclones, flooding, and mudslides (UN OCHA, 2012). According to the Rakhine Inquiry Commission Report (2013), the approximate total population of the state is 3.18 million, consisting of Buddhists (70%), Muslims (29%), Christians (0.75%), and Hindus and Animists (0.25%). Rakhine State is historically the homeland of the Rohingya ethnic minorities, who have their own language and culture (Ullah, 2011). The Rohingyas are marginalized socioeconomically, both locally and nationally and are excluded from Myanmar's social and political arena (Kipgen, 2014).

**Fig. 2:**
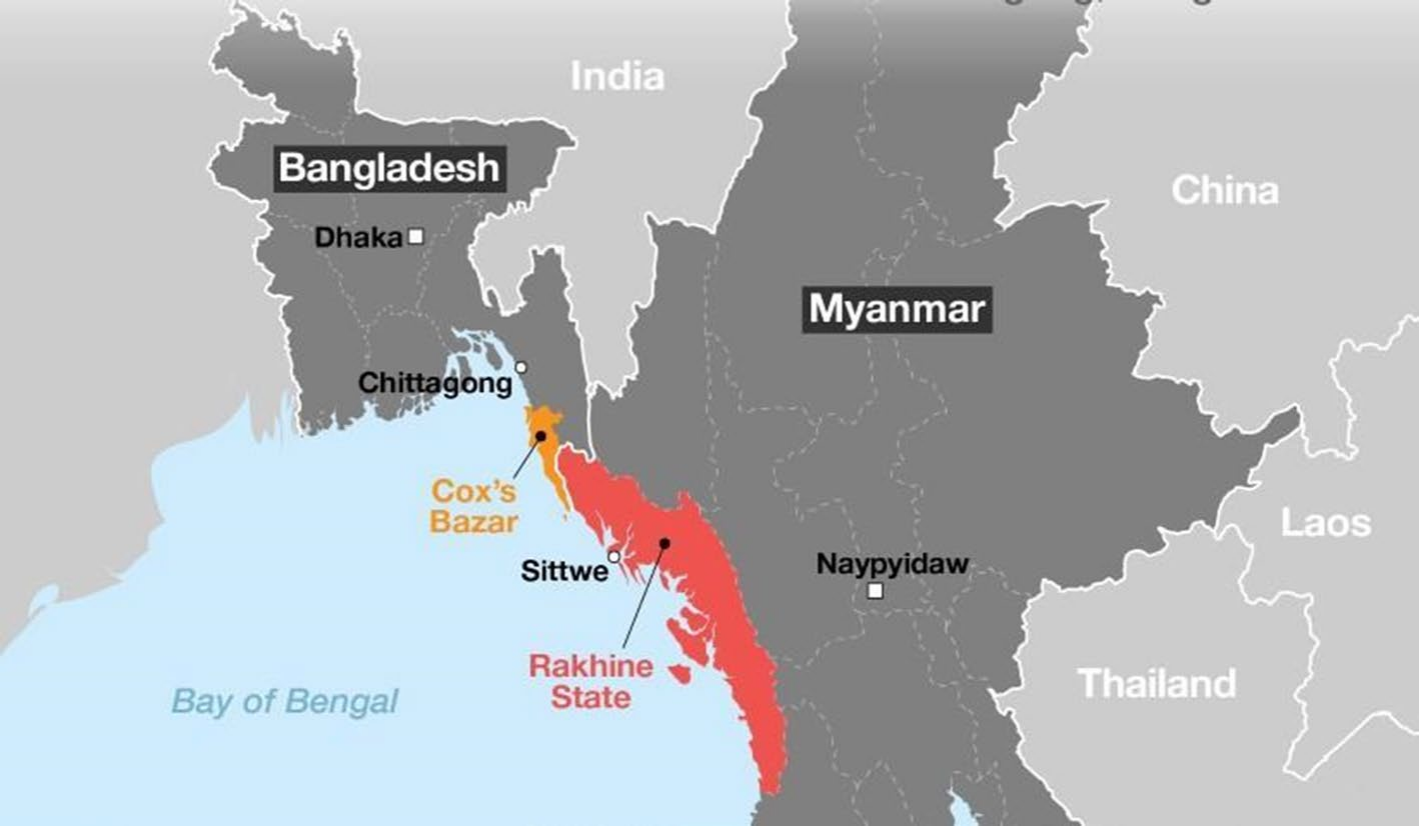
Myanmar nationals forcibly displaced to Bangladesh (DGHS, 2023). *Note*: Map showing locations of Rakhine State in Myanmar and Rohingya refugees in Bangladesh. *Source*: http://dashboard.dghs.gov.bd/webportal/pages/controlroom_rohingya.php

The Rohingya is a mixed ethnicity. Around the beginning of the 1950s, the name *Rohingya* became widely used; however, it is believed that the Muslim communities living in Rakhine State (historically called Arakan) have resided there since before the emergence of the Mrauk-U dynasty (1430–1785) of the Arakan Kingdom (Neimoto, 2005). Ethnic tensions increased when the British occupied Arakan and Tenasserim after the First Anglo-Burmese War (1824–26). During the British colonial occupation, the workforce was brought in from various parts of the Indian subcontinent, and many of these imported workers settled down and married local women (Mukherjee, 2019; Sadan, 2008).

The confrontation between Muslims residing in the northwestern part of Rakhine State and Buddhists, who are the majority in the central and southern Rakhine State, grew tenser due following the immigration of Indian workers, which included some Muslim workers from Chittagong, the southeastern part of Bangladesh (Coyle, Rahim, & Jainul, 2020). Chittagong has geo-political and historical links with Arakan, currently known as Rakhine State. However, for administrative reasons, British officials classified this latter group as Chittagonian or Mahomedan (Bowser, 2021). The migrant labor force, which dramatically changed the region’s demographic characteristics, including land ownership, co-existed with the local people but without forming a cohesive society (Chaudury & Samaddar, 2018), and unfortunately, neither the colonial government nor the local communities made any effort to integrate them.

Historically, these ethnic conflicts have been rooted in control over local resources and job opportunities, as the population growth rate is higher among the Rohingya minorities. The Buddhist population’s fear, that the Rohingyas will outperform them, feeds ethnic and religious conflict (Chaudury & Samaddar, 2018; Jones, 2017). This confrontation worsened during the Japanese occupation (1942–45), when Japan armed the Buddhist Arakanese to fight the British and the British used Muslim Arakanese forces in a counterattack. The ethnic conflicts and clashes between Buddhist and Muslim communities did not end even after Burma’s independence in 1948 (Ware, 2014; Egreteau & Mangan, 2018). 

After World War II, the Rohingya communities established autonomous areas in the northern part of Rakhine State, where they lived as citizens with full and equal rights (International Crisis Group, 2013). Later rebellious attempts on their part were defeated and control taken by the central government in those autonomous areas. However, attempts to build a stable and peaceful coexistence between Muslims and Buddhists in Arakan ended after Ne Win's coup in 1962, which transformed Burma into a strongly centralized socialist state under the control of the Army (HRW, 2002).

Political suppression arose in 1962 with policies that denied citizenship to the Rohingya population in Rakhine State. With the enactment of a revised citizenship law in 1982 excluding the Rohingya from a list of 135 ethnic groups living in Myanmar (Abdelkader, 2014), the Rohingya ethnic group became stateless (Gibson, James, & Falvey, 2016). Violence against them was reinforced by Operation Nagamin (Dragon King) in 1977, aimed at addressing the alleged immigration and inter-communal conflict, which forced over 200,000 Rohingyas to flee to neighboring Bangladesh (Doctors without Borders, 2022; Islam et al., 2022). However, many later returned home and remained isolated without basic human rights, including citizenship. In 1991–92, the government of Myanmar’s security policies and actions against the Rohingyas again forced them to flee to Bangladesh, with almost 200,000 later returning home with the assistance of UNHCR (Save the Children, 2018b) (see Table 1).

**Table 1.** Number of Rohingya refugees who entered Bangladesh in different periods (adapted from Shohel et al., 2023a)

Year	Number of refugees (approx.)	Source(s)
1942	22,000	HRW (2000)
1977–78	200,000	MSF (2022), Islam et al. (2022)
1991–92	250,000	HRW (2000), Kiragu et al. (2011)
2016	80,000	HRW (2018)
2017	700,000	Reid (2021)
2018	11,432	HRW (2018)

Communal violence and internal displacement erupted again in June 2012, when many Rohingyas were forced into refugee camps in Rakhine State (Buchanan et al., 2020). Communal violence reappeared in October 2012, triggered by the suspected rape and murder of a Buddhist woman, which led to the killing of ten Rohingya pilgrims (Jones, 2017). During this period, 140,000 Rohingyas were displaced (Lewis, 2019) and over 10,000 private, public, and religious properties were destroyed. This aggression against the Rohingyas was accompanied by a massive loss of livelihood (UN OCHA, 2013).

By the end of 2015, almost 25,000 of the displaced Rohingyas were either returned home or relocated with the support of the Rakhine State Government and the international community. However, a large proportion of them remained as internally displaced persons (IDPs) in restricted, overcrowded camps in coastal areas with very limited potential for economic activity and services that were vulnerable to natural disasters (UNHCR, 2021).

In October 2016, militant attacks on police stations in the northern Rakhine State resulted in nine police officers dead. In response, the Myanmar military sealed off the area and carried out operations targeting the militants. These operations victimized the entire Rohingya civilian population. On 25 August 2017, militant attacks on police posts in Maungdaw and Buthidaung resulted in 71 insurgents and 12 security forces dead. Since that date, almost 655,000 Rohingyas, including more than 360,000 children, have sought refuge in Bangladesh. Thousands have been killed, including women and children; people have been beaten and tortured; girls and women raped; and houses and other properties burned (Mahapatro, 2021). The Rohingyas have been accommodated in both registered and unregistered refugee camps, makeshift settlements, and spontaneous host communities in Bangladesh, often in inhumane conditions (Hassan et al., 2018). The Institute on Statelessness and Inclusion (ISI) has been deeply concerned about the safety and security these refugees (ISI, 2017).

The Global Humanitarian Overview 2022 (UN OCHA, 2022) has described the conditions in the camps as follows:

The world’s biggest refugee camp battles regularly with the onset of monsoon rains. The Bangladeshi Government and humanitarian organizations on the ground work hard to minimize the risks from landslides, flash floods, waterborne diseases, and ultimately, loss of life. Thousands of people face dire circumstances, as conditions in the camps are expected to dramatically worsen with the onset of heavy rains.

The government of Bangladesh has relocated some Rohingya refugees to a remote island called Bhasan Char in the Bay of Bengal (Gazi et al., 2022) despite urging from humanitarian organizations not to do so, as the transfer of refugees requires independent assessment and the refugees’ informed consent. The Rohingya refugees relocated to this island have complained that they are denied freedom of movement and have no access to a sustainable livelihood or education (Human Rights Watch, 2020). Very little is known thus far about the relocated Rohingya refugees in Bhasan Char.

## Methodology

This article is based on existing literature collected from different sources. A systematic approach (Jesson et al., 2011) was followed in collecting documents, i.e. academic and grey literature, on the Rohingya crisis and its impact on the displaced Rohingya children living in refugee camps in Bangladesh. The keywords “Rohingya”, “refugee”, “children”, “childhood”, “education”, “camp”, and “Bangladesh” were used in the search tools Google, Google Scholar, Mendeley, Semantic Scholar, Social Science Research Network, and Research Gate. Both freely available academic and non-academic published and unpublished documents written in English were collected. Grey literature such as reports, working and conference papers, newsletter and newspaper articles, government documents, and agency documents were also included in this review. Selected literature was explored to analysis secondary data and thematic analysis was used to complete the review.

## Childhood, children’s rights, and education

After exploring the context of the Rohingya crisis, this article paints a wider picture of childhood experiences in the camps. This section is organized around core principles of the United Nations Convention on the Rights of the Child, along with the feelings and opinions of the Rohingya refugees drawn from secondary sources.

### Stolen and lost childhood in the refugee camps

Children are considered to be at a stage of life entitling them to special care and assistance (Archer & Oppenheim, 2021). Children must be given the opportunities to be fully prepared to live in society and allow them to enjoy the right to rest, play, and engage in recreational and developmental activities. That is not the case for the Rohingya children living as refugees abroad or as internally displaced individuals (IDPs) in Myanmar. Many Rohingya children living in the world’s largest refugee camp, in Cox’s Bazar, Bangladesh, have been deeply traumatized by their experiences (Oxfam International, 2021) and require mental health counseling and support to cope with their trauma and collective memories (Shohel et al., 2022b).

This article bases its view of “a desirable childhood” on the UN Convention on the Rights of the Child (UN General Assembly, 1989). Core childhood rights enumerated in the preamble to the convention and also mentioned in the Universal Declaration of Human Rights and in the Convention related to the Status of Refugees (UN General Assembly, 1951) include a secure family environment; an atmosphere of happiness, love, and understanding; freedom; dignity; equality; and legal protection.

The Rohingya crisis has placed the lives of the displaced and desperate Rohingya children in limbo. As noted, the Rohingya refugee children have been exposed to trafficking, child marriage, exploitation, and abuse (Hutchinson, 2018). Already stateless, they have also become rightless and face an uncertain future. It seems they are no one’s responsibility, and the possibility of their returning home is uncertain (Yee, 2017). In the eyes of the international community and in the context of the twenty-first century’s sustainable development goals (SDGs), ensuring equality, equity, and social justice for these children is paramount. Without adequate access to basic human needs, including learning, and without hope, these futureless children might easily become involved in anti-social behavior and criminal activities, like arms and drug smuggling (O’Driscoll, 2017).

The Rohingya children in the refugee camps in Bangladesh are missing the opportunity to have a decent and enjoyable childhood. They have minimal access to education, which is vital to every child’s development and even more critical in the current situation. These children live with adult responsibilities, helping their families and community and sometimes placing school in second place. A lack of money and the absence of a clear future or vocational path leads many to leave school early to start families as they see their elementary or basic education is useless. Their frustration and fears are reflected in a poem titled “Fleeing the homeland”, by a 16-year-old Rohingya child named Omar:

- No roof to take refuge
- Being blurred the way to home
- No face to see
- No word to speak
- No ground to play
- Humans helped humans
- Million thanks aren’t enough
- Billion prayers aren’t adequate
- Having no paddle
- Can’t find any shores
- Uncountable helping hands
- Pulled me to the shores
- Now I have a roof
- Faces with welcoming smiles
- Words to speak
- Pens to write
- Songs to sing
- Now I am a player general
- The way to home becomes brighter (Lateef, 2020).

The lack of access to higher education for refugees (Shohel et al., 2021a) especially no access to higher education for the Rohingya refugees (Shohel et al., 2023b), high teacher turnover, restrictions on infrastructure, and scarce resources, among other factors, have limited the opportunities of the displaced Rohingya children (Shohel, 2022). If their curiosity, ambitions, and educational needs are not satisfied, their potential will be un-nurtured and wasted. They have aspirations, just like other children. Some want to be politicians, some wish to teach human rights, some want to be doctors, engineers, lawyers, journalists or writers. A significant number of these young people work very hard, seizing every opportunity they are given and remaining focused on their goals in the face of uncertainty.

### Children’s rights

Children’s rights are acknowledged and secured by the 1989 UN Convention on the Rights of the Child. Bangladesh signed and ratified this Convention in 1990 and is therefore bound to implement its vision to protect all children equally. However, Bangladesh did not sign the 1951 Convention Relating to the Status of Refugees or its 1967 protocol on adherence to the protection of the rights of asylum-seekers and refugee children and their access to essential services. The Convention on the Rights of the Child recognized that “children are the holders of their own rights and are therefore not passive recipients of charity but empowered actors in their own development”. However, being a “recipient of charity” or being “empowered actors in their own development” is not determined only by the international declaration of intentions. Beyond survival, the Rohingya children want to play and learn to become successful in their adult lives (UNICEF, 2019b).

The Rohingya children living in the camps do enjoy some of the opportunities their families were lacking, such as freedom of speech, protected in Article 13 (children have the right to find and share information and opinions without discrimination). This right was denied to children living in Rakhine State. Now, inside the camps in Bangladesh, children are allowed and even encouraged to share their views and opinions. Article 19 states that children have the right to be protected from suffering physical or mental violence or any other type of abuse and that countries should adopt measures accordingly.

The international community strongly stresses the right of children to play (Article 31). With regard to children’s development and happiness, this is perhaps one of the most important rights of the UN Convention and something children in the camps have many opportunities for.

Article 32 states that children have a right to be protected from economic exploitation and from performing work that is hazardous or interferes with their education, yet it is not uncommon for adolescents to join their families in illegal work outside the camp. Their working hours are often long and hard, and they are poorly paid. Each time young people step outside the camp’s parameters, they risk arrest by law-enforcement authorities.

Myanmar signed and ratified the UN Convention on the Rights of the Child (CRC) in 1991 and recently enacted a Child Rights Law that is supposedly in accordance with the CRC. According to the CRC, children have the right to acquire nationality and citizenship, and no child should be stateless. This is unfortunate that Myanmar has been denying this right of the Rohingya children since 1982 which is against the CRC.

The Rohingya children know that Bangladesh is not their country and that local communities do not always welcome them. Two reports by Human Rights Watch titled *“Are we not human?”: Denial of education for Rohingya refugee children in Bangladesh* (2019) and *“Bangladesh is not my country”: The plight of Rohingya refugees from Myanmar* (2018) clearly portray the situation of the Rohingya children in the refugee camps.

The above-mentioned reports, based on consultation with the Rohingya refugee children, show that their childhood has been profoundly interrupted due to conflict and violence (Plan International, Save the Children International, & World Vision International, 2018). According to the *Childhood interrupted: Children’s voices from the Rohingya refugee crisis* report:

“Many of these children have previously reported fleeing burning homes, targeted arson and widespread violence. Most children arrived with no material possessions, but the clothes on their backs, and thousands now find themselves as the head of their households” (Plan International, Save the Children International, & World Vision International, 2018).

Unfortunately, the Rohingya children do not enjoy the legal safeguards articulated in the CRC and Child Rights Law. It must be emphasized again that children, especially girls, are very vulnerable to prostitution, violence, and trafficking (including sex and manual work). Disabled children are also vulnerable and require special attention and support (Shohel et al., 2022b). During the reported interviews mentioned above, the children said that they want to be healthy and learn and play while they are still in refugee camps. Accordingly, they should be given opportunities to enjoy childhood and develop as individuals.

Despite violations of the United Nations CRC (1989), the Rohingya refugee children retain hope for a better future and believe one day they will return to their villages in Myanmar.

### Right to education

Education is recognized as one of the fundamental human rights under international and regional human rights law and in many international documents (McCowan, 2013), including the Universal Declaration of Human Rights (1948) [Article 26], the Convention Relating to the Status of Refugees (1951), the Declaration of the Rights of the Child (1959) [Principle 7], the International Covenant on Civil and Political Rights (1966), the International Covenant of Economic, Social and Cultural Rights (1966) [Articles 13 and 14], the Declaration on Social Progress and Development (1969) [Articles 10 and 11], the Convention on the Elimination of All Forms of Discrimination Against Women (1979), the CRC (1989) [Articles 28, 29, and 32], and the Dakar World Education Forum Framework for Action (2000). As enunciated in these documents, every child regardless of background has the right to an education and a decent childhood to prepare themselves for a better future.

The right to education includes learning about rights and responsibilities in order to become a good citizen; a congenial, high-quality teaching and learning environment; freedom from violence, bullying, and harassment; respect for individuality and diversity; and full participation of all children. Education as a human right is vital for understanding other human rights; it encompasses civil, political, economic, social, and cultural rights (UNCHR, 1995). Therefore, the role of education is given special consideration in Articles 19, 23, 24, 28, 29, 32, and 33 of the Convention. Articles 28 and 29 state that every child should have equal access to educational opportunities, regardless of wealth or other factors. According to the CRC, education should nurture the development of each child’s personality, including their talents and mental and physical abilities, to their fullest potential. Article 31 emphasizes that every child has the right to learn about their own cultural identity, language, and values. The Convention also stresses the need for compulsory and free primary education with regular attendance and accessibility to secondary and higher education.

Education promotes the healthy development of present and future generations; it is essential for unlocking the potential of every individual, for enjoying the full range of human rights, and for learning to respect the rights of others. Education is essential to exercising one’s agency as “the concept of human rights is inseparable from their role in international political practice” (Dum, 2016). Education uplifts those who are marginalized socioeconomically and culturally by empowering them to pull themselves out of poverty and acquire the means to participate fully in their communities.

Unfortunately, displaced Rohingya children living in Bangladeshi refugee camps have very limited access to education (Babu, 2020; Shohel, 2022). Hammadi (2020) aptly noted that:

However, what is more important for us to see is the consequence of growing a generation without education, a generation that will not be able to speak up for themselves, speak against the violation of their rights, enjoy the benefits of an active and enlightened mind, or lift themselves out of their difficult situation.

The results of a survey completed in December 2018 of 180,000 Rohingya children ages 4 to 14 showed the extent of the need for education (UNICEF, 2019a). Survey results indicate that the majority of the Rohingya children did not have regular access to education in Myanmar. More than 90% were shown to have learning competencies from the pre-primary level to grades 1 and 2. Just 4 percent were at grade levels 3 to 5, and 3 percent at grades 6 to 8. There is an urgent need to provide opportunities for adolescents, as only 7 percent of those 15 to 18 years old are accessing education. However, 45,000 adolescents aged 15 to 18 were targeted for programs providing foundational literacy, numeracy, and relevant vocational skills (UNICEF, 2019a).

In January 2019, UNICEF led the development of a newly structured learning program known as the Learning Competency Framework and Approach (LCFA) (UNICEF, 2019a; Shohel et al., 2023a). The LCFA defines learning competencies (along with the approach to achieving them) comparable to those children would achieve through a formal school curriculum. The learning framework covers the following subjects: English and Burmese language, mathematics, life skills, and science across levels 1 to 5. The Education Sector in Cox’s Bazar has provided informal education opportunities to 324,000 Rohingya children aged 4 to 14 years based on the LCFA (Reid, 2020). In addition, over 10,000 Rohingya adolescents aged 15 to 18 years have received literacy, numeracy, life-skills, and vocational-skills training. The children of the UNICEF-supported learning centers are now enrolled based on their competency level, whereas previously they were placed in temporary learning centers according to their age.

Recently, the government of Bangladesh has agreed to allow the Rohingya refugee children access to formal education under UNICEF leadership (Shohel et al., 2023a, b). This is a clear indication of the commitment of the government of Bangladesh to ensure access to learning for the Rohingya children and adolescents and equip them with the right skills and capacities for their future and their return to Myanmar when conditions allow. The United Nations and humanitarian organizations have welcomed Bangladesh’s decision to expand access to education for the Rohingya children and adolescents living in the Cox’s Bazar camps (The Financial Express, 2020).

**Fig. 3:**
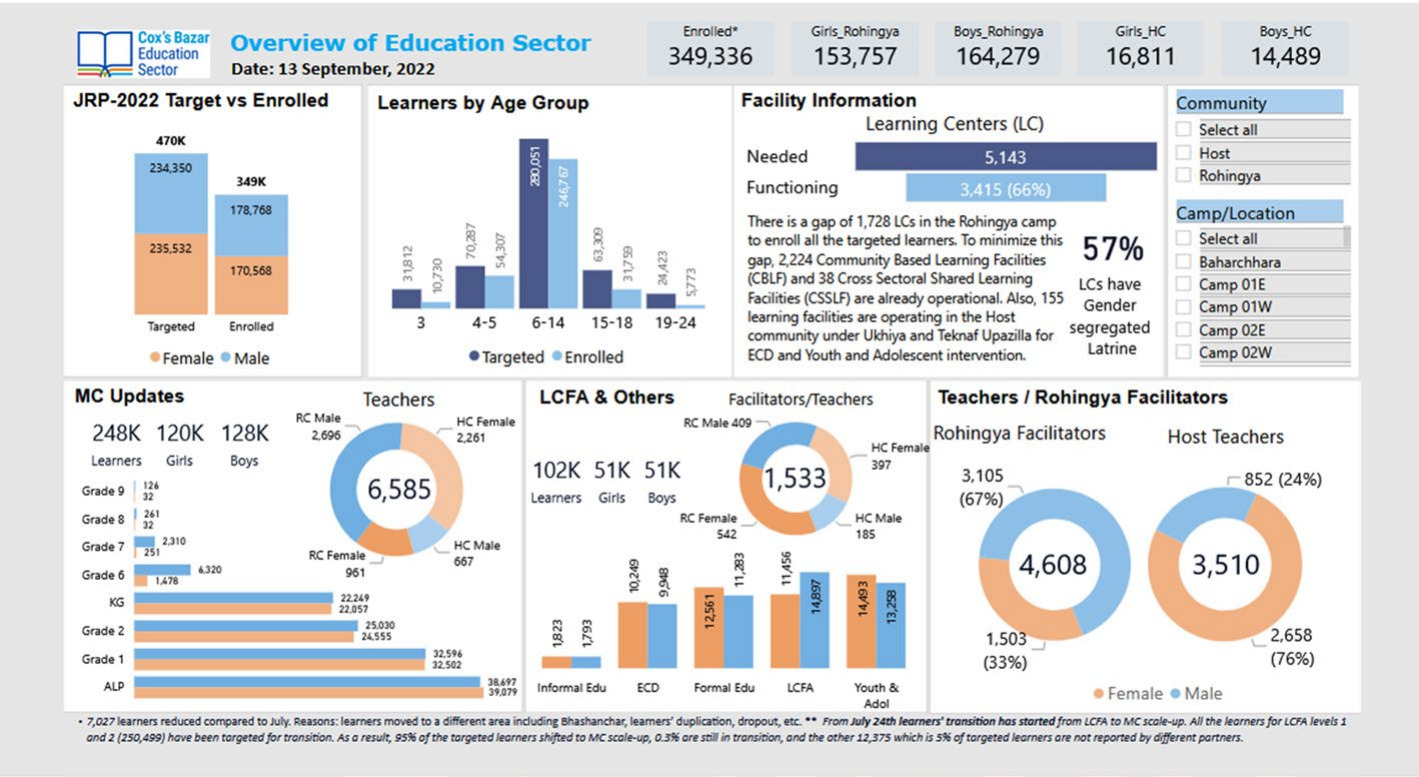
Overview of Education Sector humanitarian support in Bangladesh. *Source*: https://www.humanitarianresponse.info/en/operations/bangladesh/education. *Note*: *Education Secto*r is the umbrella term for the joint efforts of NGOs in the field of education. It is widely used by development organizations working in Cox’s Bazar.

In line with the government of Myanmar’s decision, the Education Sector for the Humanitarian Response in Cox’s Bazar piloted the introduction of the school curriculum of Myanmar in the Rohingya refugee camps starting in November 2021, initially targeting 10,000 Rohingya students in grades 6 to 9 (Shohel et al., 2023a). However, considering the current enrolment, reaching the target would be difficult (see Figure 3). The use of the school curriculum of Myanmar will be expanded to other grades in different phases. According to the UN, these efforts can help accelerate an expansion of education, particularly to older children, make the content of education more relevant for refugees, and allow UNICEF to meet the educational wishes of the Rohingya people more comprehensively (UNICEF, 2022). When conditions become conducive and refugees can return to Myanmar in a safe, dignified, and sustainable way, these efforts will also help children reintegrate into the mainstream education system of Myanmar.

Currently, one-third of the 416,000 school-age Rohingya children (3–18 years old) are still not accessing basic education (UNICEF, 2019). A generation of the Rohingya children and young people cannot be left without the education and skills needed to build a life for themselves. If they become self-sustaining, their communities will also become self-sustaining and flourish. Education as a tool of individual and community development will help them rebuild their lives after returning to their home in Myanmar.

## Recommendations

The complexity of the Rohingya refugee crisis is fundamentally in the hands of the government of Myanmar, as the state denies their citizenship. Although other ethnic minorities are in conflict with the majority of Burmese in Myanmar, the Rohingya minority’s situation is unique. While other ethnic minorities demand autonomy and equal rights under a federal setup, the Rohingya struggle to be recognized as one of the ethnic groups of the country (Kipgen, 2013). For peace and communal harmony, relationships between Rohingyas and Rakhines and with the people of Myanmar in general need to be improved. Considering the history and geopolitics of the region as well as the unique nature of the history of the Rohingya ethnic group, challenges of reconciliation and political integration must be addressed through multilateral agreements and efforts.

A strong political desire from the international community, the government of Bangladesh and the government of Myanmar needs to be demonstrated in decision-making for the Rohingyas to return to Myanmar (Neimoto, 2005). A reconciliation program will have a chance to succeed only when the Rakhines and the Rohingyas are willing to compromise on their differences, respect each other’s identity, and acknowledge and accommodate each other's culture. For any genuine reconciliation to be realized, the government and general public of Myanmar must be ready to embrace the Rohingyas as part of their larger society. Attempts to achieve long-term solutions by ignoring the crux of the problem namely, political integration are unlikely to bring real peace and stability to Rakhine State of Myanmar. Therefore, practical supports and solutions must be in place to reduce the burdens of Bangladesh and international community to support the Rohingya children in refugee camps.

Under the reality of uncertainty, it is essential to assist development workers to support the Rohingya refugee children in need. The initial response to the need for educational opportunities for the displaced Rohingya children should focus on expanding and improving existing learning opportunities. Through UNICEF leadership, all Rohingya children could be ensured equitable access to education. The support initiatives could be further strengthened by standardising the education in emergencies (EiE) response and providing psychosocial support through various initiatives and interventions (Shohel et al., 2022b). These refugee children's experiences must be understood to support their needs, help them overcome challenges, remove their fears, and enhance their hopes for the future through refugee-centred education (Naidoo, 2009). This gives children a great sense of belonging but prevents them from having a worry-free childhood as their future is uncertain. Initiatives should be taken to stress and respect the differences between childhood and adulthood.

Benefits such as extra rations or a “child benefit” should be used as incentives to keep the Rohingya children in school and discourage them from going to work. The presence of NGOs has had many benefits for refugee children, such as giving them the time and space to play and freely express themselves, be creative, and access information that will improve their health and well-being. During the interviews mentioned earlier, the Rohingya refugee children expressed their eagerness to make the most of every opportunity they have, and despite the unfavourable conditions of living in refugee camps, they are unwilling to become passive recipients of charity. They have shown their resilience, tenacity, and determination in coping with incredibly difficult circumstances.

Learning opportunities and facilities need to be scaled up from the current response level to enable more Rohingya children and youth to exercise their right to education. Innovative education delivery methods must be used, and inter-sectoral collaboration should be encouraged to increase the effectiveness of available resources, such as using temporary learning centers as multifunctional spaces and integrating learning in other facilities for children in response to congestion and land availability problems.

Through blended-learning approaches, flexible learning models could provide a safe and inclusive environment in which quality interventions lead to effective learning (Shohel et al., 2022b). Therefore, as part of the process of healing and peace-building efforts, teaching life skills and global citizenship education should be a key strategy going forward (Shohel et al, 2023b). Innovative strategies such as providing e-learning opportunities are needed to ensure that vulnerable groups have access to materials that meet their learning needs (Shohel et al., 2021b & 2022a), especially for adolescent girls, child laborers, children with disabilities, and child-headed households.

The quality of education must be improved by developing teaching and learning strategies that are tailored to the varying needs of the Rohingyas and host communities. Initiatives are required to promote durable solutions through advocacy and cooperation with education authorities. It is important to ensure close supervision for enhancing school attendance and enjoyable learning environment. Communities must be involved in overseeing school enrolment, attendance, retention, and educational outcome; in ensuring participation; and in encouraging parental engagement in children's education.

Given the historic discrimination against the Rohingya people and limited access to education for the Rohingya children in Rakhine State, the availability of the qualified Rohingya applicants for available positions within the humanitarian organizations is often limited. For the same reason, there is a lack of teaching staff. Therefore, teacher retention and capacity development should be addressed through supportive supervision, mentoring, coaching, and the provision of professional development opportunities in pedagogical approaches, psychosocial support, subject-based instruction, and life skills. Sports, recreational, and life-skills activities must be integrated into a curriculum that focuses on developing the community in the context of camp life and beyond. However, individuals’ capability enhancement activities should be offered, with a special focus on pre-adolescent boys and girls, to promote social cohesion and build resilience.

Robust adult education projects for those aged 16 to 24 years could be undertaken using a variety of delivery methods including digital, face-to-face, peer-to-peer, as well as indoor games and outdoor sports for development to ensure that young refugees acquire the necessary skills for productive engagement. Critical thinking, problem-solving, and empathy should be strong elements of such a literacy initiative. Given the social norms currently prevalent among the Rohingyas, it is critical that girls and young women are especially targeted so they can be empowered to become self-dependent. Given the gender norms, there is also a need to ensure that girls and young women can participate in the learning process as learners and facilitators through targeted approaches.

More than 52% of newly arrived refugee children and youth since August 2017 are girls. Considering the high drop-out rate among girls, improved gender mainstreaming and targeted interventions are needed for adolescent girls. This includes creating a safe learning environment; ensuring access to education facilities, including gender-sensitive premises; recruiting female teachers; and linking to cash-based interventions and menstrual hygiene management.

Low household-income levels force the Rohingya children to engage in income-generating activities such as domestic work to support the family rather than going to school (Crabtree, 2010). The Rohingya refugees remain reliant on humanitarian assistance. There are incidents of families selling personal items for cash to supplement assistance and seeking other ways to bring in cash. The Rohingya refugees are not permitted to work within host communities. Unofficially, they are regularly hired for day labour and the informal employment sector at consistently lower rates than Bangladeshi day laborers. As a result, competition in the local labour market has led to tensions and conflicts between the two communities.

Synergies in multi-sectoral interventions should be explored to ensure they complement efforts to build family capacities to achieve greater self-reliance by acquiring livelihood skills and the attendant soft skills. Dedicated programs should be developed and implemented by focusing on life skills, vocational training, and basic literacy and numeracy based on real-world requirements (setting up micro-enterprises, family-based production of food and non-food items, and access to e-knowledge networks) to optimise family outcomes with regard to income and social well-being. Such a strategy could significantly improve knowledge and skills and reduce the tendency to resort to negative coping mechanisms. These kinds of interventions will benefit about 20% of youth between ages 15 and 24 among refugees and host communities. Considering the fluid and unsettled life in the camps and settlements, this set of cross-sectoral interventions is critical to reducing the risks of trafficking, drug abuse, early marriage, and hazardous and exploitative work inside and outside the camps (Babu, 2020; Shohel, 2022).

Opportunities should be created, prompted by the situation of the Rohingya people, to carry out research that brings together a novel combination of disciplines to effectively inform thinking on human rights and social justice. Academic researchers must be engaged in developing ideas and exchanging expertise so that research-informed policy and practice will be in place to tackle the current “complex emergencies” in relation to fulfilling the post-pandemic needs and addressing the impact of long-term refugee crisis. For example, interdisciplinary research networks might enhance the impact of development initiatives and interventions on aid assistance and on practitioners' supports to the refugees on the ground.

Academics from Bangladesh and Myanmar need to be engaged in academic dialogue, research, and discourse that contributes to achieving SDGs for both countries (SDGs 2, 4, and 10.7; also significant SDGs are 3, 5, and 6), including migration and refugee crises that mostly impact socioeconomic and ecological development negatively. There is a lack of academic skill and expertise to carry out interdisciplinary research to solve the global challenges of migration and refugee crises, especially in Bangladesh and Myanmar. As suggested in the grey literature, there is a need for livelihood related interventions to provide social protection and safety-net schemes for vulnerable refugee children as well as support for their hosting communities. The outcomes of any collaborative research in these areas will better equip development workers and academic researchers to design, develop, and implement a framework to bring change to the lives of these displaced Rohingya children.

Academic collaboration and research are needed to assist practitioners on the ground in effectively supporting the Rohingya refugee children, ensuring their human rights and providing them with quality education and health care. Interdisciplinary research activities should be in place to assist in developing a holistic framework that can be adopted by both governmental organizations (GOs) and nongovernmental organizations (NGOs) to support the education, mental health, and well-being of the displaced Rohingya children living in Bangladeshi refugee camps. Within such a framework, these refugee children could receive basic health education and acquire skills to engage in income-generation activities, so they do not become a burden on anyone. At the same time, the framework should engage them in a constructive educational process to overcome their traumatic experiences, the loss of their loved ones, the misery of life in refugee camps or temporary shelters, and other difficulties and create opportunities for them to develop their full potential as human beings. Doing so will also prepare them to be rehabilitated when the time comes for them to return to their homes in Myanmar or migrate to a third country.

## Conclusion

The international community has an enormous moral and ethical obligation to address the issues related to the stolen and lost childhood of the Rohingya children and to safeguard the human rights of these vulnerable, stateless, displaced children (Save the Children, World Vision and Plan International, 2018; Shohel, 2022). The government of Bangladesh has been exploring repatriation, relocation, and third-country settlement options for the Rohingya refugees. However, the Rohingya crisis is a complex issue, related not only to religious or ethnic conflict but also to numerous geopolitical and economic interests (Bepler, 2018; Neimoto, 2005). Bilateral agreements between Bangladesh and Myanmar and multilateral agreements with Myanmar and other countries are needed for a permanent solution (Ahmed & Mohiuddin, 2019). In every humanitarian crisis, humanitarian organizations have a responsibility to be accountable and listen to the communities they support, in accord with their commitment to the Core Humanitarian Standard (CHS Alliance, Group URD, & the Sphere Project, 2014).

There are significant concerns about returning of the Rohingya refugees to their homes as well as uncertainty with regard to their willingness to return to Myanmar, where they have been victims of ethnic cleansing and genocide (Mohajan, 2018; Save the Children, 2018b; Zarni & Cowley, 2014). The international community, including the UN and neighboring countries, must put pressure on the government of Myanmar to find a permanent solution to the conflict, to take the Rohingya refugees back, assist them to settle down, and build peace across the country, especially within Rakhine State, so that the Rohingya community, as an ethnic minority, can live in harmony with the majority Buddhist Rakhine community.

The future of the Rohingya children currently depends on both the international community's support to the Rohingya refugees and its negotiations with the government of Myanmar to repatriate them (ISI, 2017). The over-reliance on NGOs and external aid does not resolve difficulties of access to livelihood and education. A more sustainable, community-led system is needed within the refugee camps (Shohel et al., 2023b). Autonomy would ensure that even if funding and external support run out, these communities would be able to keep moving and growing by using their own resources. At the same time, in addition to financial support, a range of tools are necessary for independent and sustainable community development. Greater access to resources such as the internet, technology, information, quality teachers, recognized academic certification, and university scholarships are vital to the advancement of the whole community. It is a global responsibility to improve access to these resources and opportunities for the Rohingya children and their families to have their own dignified lives.
